# Genomic Analysis of Milk Protein Fractions in Brown Swiss Cattle

**DOI:** 10.3390/ani10020336

**Published:** 2020-02-20

**Authors:** Lucio Flavio Macedo Mota, Sara Pegolo, Vittoria Bisutti, Giovanni Bittante, Alessio Cecchinato

**Affiliations:** Department of Agronomy, Food, Natural Resources, Animals and Environment (DAFNAE), University of Padova, Viale dell’ Università 16, 35020 Legnaro, Italy; flaviommota.zoo@gmail.com (L.F.M.M.); vittoria.bisutti@unipd.it (V.B.); giovanni.bittante@unipd.it (G.B.); alessio.cecchinato@unipd.it (A.C.)

**Keywords:** genetic parameters, genetic correlations, caseins, whey proteins

## Abstract

**Simple Summary:**

Milk protein fractions are hugely important in the dairy industry because of the key role they play in milk technological properties. The selection of cows for milk protein fractions may, therefore, improve both the nutritional and technological characteristics of milk, and, consequently, the processing efficiency and value of the dairy product. This study estimated the genetic parameters of the major milk protein fractions (four caseins, and two whey proteins) determined variously as: (i) milk content (g/100g milk), (ii) percentage of milk nitrogen (%N) and (iii) daily yield (g/d) in Brown Swiss dairy cattle. The results showed that the (co)variances and genetic parameter estimates differed according to how the proteins were measured. These results provide useful information for developing selection strategies in dairy cattle breeding programs aimed at improving both the nutritional and technological properties of milk.

**Abstract:**

Depending on whether milk protein fractions are evaluated qualitatively or quantitatively, different genetic outcomes may emerge. In this study, we compared the genetic parameters for the major milk protein fractions—caseins (α_S1_-, α_S2_-, β-, and к-CN), and whey proteins (β-lactoglobulin, β-LG; α-lactalbumin, α-LA)—estimated using the multi-trait genomic best linear unbiased prediction method and expressed variously as milk content (g/100g milk), percentage of milk nitrogen (%N) and daily yield per cow (g/d). The results showed that the genetic parameter estimates varied according to how the milk protein fractions were expressed. Heritability estimates for the caseins and whey protein fractions expressed as daily yields were lower than when they were expressed as proportions and contents, revealing important differences in genetic outcomes. The proportion and the content of β-CN were negatively correlated with the proportions and contents of α_S1_-CN, α_S2_-CN, and к-CN, while the daily yield of β–CN was negatively correlated with the daily yields of α_S1_-CN and α_S2_-CN. The Spearman’s rank correlations and the coincidence rates between the various predicted genomic breeding values (GEBV) for the milk protein fractions expressed in different ways indicated that these differences had a significant effect on the ranking of the animals. The results suggest that the way milk protein fractions are expressed has implications for breeding programs aimed at improving milk nutritional and technological characteristics.

## 1. Introduction

Milk protein fractions affect milk-derived products in terms of quality, milk coagulation ability and cheese yield, and therefore have a marked economic impact on the dairy industry [1]. The major milk protein fractions in milk are four caseins (CN), α_S1_-, α_S2_-, β- and к-CN, and two whey proteins, α-lactalbumin (α-LA) and β-lactoglobulin (β-LG), which together account for approximately 90% of total milk protein fractions [2] and play a key role in determining milk technological properties. Changes in milk protein composition can affect curd formation and cheese yield [3]. Milk protein fractions could therefore be included in the selection criteria in dairy cattle breeding programs aimed at improving cheese-making-related traits, assuming use of a rapid and effective method such as Fourier Transform Infrared (FTIR) spectroscopy for the determination of these traits at the population level [4]. Modifying the milk protein composition might offer also an opportunity to improve the nutritional value of milk [5].

From the animal breeding perspective, increasing the potential of cows to produce milk with greater amounts of casein could in turn increase the cheese-making aptitude of the milk [1,6]. However, if these traits are to be included in the selection indices of dairy cattle, thorough knowledge of their mutual relationships is required in order to design selection strategies that positively affect milk quality and cheese-making aptitude [3,7]. Several studies have reported genetic parameters for milk protein fractions expressed as percentage of total protein or as percentage of milk and investigated their mutual relationships [3,5,8,9,10]. However, to our knowledge, information regarding biological and genetic implications of using different ways of expression for milk proteins (assessed using the same analytical method, the same population and breed) in the animal breeding context is still scarce.

We hypothesized that different ways of expressing milk protein fractions (as either qualitative or quantitative data) could lead to differences not only in the genetic parameters for these traits, but also in the estimated genomic breeding values, which could in turn lead to re-ranking of the top animals. It is, therefore, crucial that these differences are taken into account when developing effective selection strategies in dairy cattle. Therefore, using a multi-trait framework we estimated the genetic parameters of milk protein fractions—four caseins (β-CN, k-CN, α_S1_-CN, and α_S2_-CN), and two whey proteins (WP: α-LA and β-LG)—in Italian Brown Swiss dairy cattle, and compared the resulting data variously expressed as g of protein secreted per day (g/d), percentage of nitrogen (%N) and g/L of milk (g/L).

## 2. Materials and Methods

### 2.1. Ethics Approval

The cows included in the current study belonged to commercial private farms and were not subjected to any invasive procedures. Milk and blood samples were previously collected during routine milk recording coordinated by technicians from the Breeders’ Association of Trento Province (Italy), and were hence certified by the local authority.

### 2.2. Phenotypic and Genotypic Information

Milk samples were collected once from 1,264 Italian Brown Swiss cows belonging to 85 commercial herds during the evening milking. Details of the animals included in the study are reported in Bittante et al. [11] and Cecchinato et al. [12]. Somatic cell counts were assessed using a Fossomatic FC counter (Foss Electric A/S) and converted to somatic cell scores (SCS) by logarithmic transformation, as proposed by Ali and Shook [13]. Milk quality traits were measured for total nitrogen, casein, and urea nitrogen (MUN) using a MilkoScan FT6000 apparatus (Foss Electric A/S, Hillerød, Denmark). The casein fractions (α_S1_-CN, α_S2_-CN, β–CN and κ-CN) and whey proteins (α-LA and β-LG) were determined by validated reversed-phase high-performance liquid chromatography (RP-HPLC), as described by Bonfatti et al. [14]. Total casein (CN) was defined as the sum of the casein fractions (β-CN, k-CN, α_S1_-CN, and α_S2_-CN), and total whey protein (WP) as the sum of α-LA and β-LG. Milk protein fractions were expressed as (i) the percentage of the total milk nitrogen content (%N), (ii) g/L of milk (g/L), and (iii) g of protein secreted per day (g/d), a measure that accounted for the cows’ milk production levels. The milk protein fractions underwent phenotypic quality control to remove observations more than three standard deviations below or above the mean of the herd; a boxplot of the phenotypic values for the milk casein fractions can be found in Supplementary Information Figure S1.

A total of 1050 cows were genotyped using the Illumina Bovine SNP50 v.2 BeadChip (Illumina Inc., San Diego, CA, USA). Genotypes underwent quality control to exclude the sexual chromosome regions. Autosomal SNP markers with minor allele frequencies (MAF) lower than 0.05, that deviated significantly from the Hardy–Weinberg equilibrium (*P* ≤ 10^−5^) or had a call rate lower than 0.95 were removed. A total of 989 animals and 37,519 SNP markers were retained in the genomic dataset.

### 2.3. Statistical Analysis

A principal component analysis (PCA) was carried out to reveal possible population substructures in the study population. The principal components were computed with the gaston R package [15], and the animals were clustered into two groups; the first two components were considered co-variables in order to correct the population effect (Supplementary Information: Figure S2).

A multi-trait genomic best linear unbiased prediction (GBLUP) model was used to infer the genetic parameters for each set of traits separately (i) milk yield and milk protein fractions (β-CN, k-CN, α_S1_-CN, α_S2_-CN, β-LG, and α-LA) expressed as g/L; (ii) crude protein content and milk protein fractions expressed as %N; (iii) milk protein fractions expressed as g/L, and iv) for the total protein, total casein (CN) and total whey protein (WP) expressed as g/L, %N and g/d, according to the following model:

y=Xβ+Za+e,

where y is the matrix for phenotypic information for the traits being investigated; β is the vector of fixed effects defined by days in milk (6 classes: class 1, less than 60 days; class 2, 60–120 days; class 3, 121–180 days; class 4, 181–240 days; class 5, 241–300 days; class 6, more than 300 days), parity of the cow (4 classes: 1, 2, 3, ≥4), and herd-date effect (n = 85), and the first two principal components as a linear co-variable to correct for population sub-structures; ***a*** is the vector of additive genetic effects; X and ***Z*** are the incidence matrices relating **y** to effects β and a, respectively.

The additive genetic and residual effects were assumed to be normally distributed: $a=\left\{a_{i}\right\}\sim MN\left(0,\Sigma_{g}⊗G\right)$ and $e=\left\{e_{\mathrm{ij}}\right\}\sim MN(0,\Sigma_{e}⊗\;I),$ where $\Sigma_{g}=\;\left[\begin{array}{ccc} \sigma_{a1}^{2} & \cdots & \sigma_{a1,n}\\ \vdots & \ddots & \vdots \\ \sigma_{a1,n} & \ldots & \sigma_{an}^{2} \end{array}\;\right]$ and $\Sigma_{e}=\;\left[\begin{array}{ccc} \sigma_{e1}^{2} & \cdots & 0\\ \vdots & \ddots & \vdots \\ 0 & \ldots & \sigma_{en}^{2} \end{array}\right]$ are the variance matrices for the additive genetic and residual effects, respectively; **I** is the identity matrix; and **G** is the genomic relationship matrix according to Van Raden [16]. The **G** matrix was constructed as follows: $G=MM^{\prime }q$ where M is the SNP matrix assuming 0, 1, and 2 for genotypes AA, AB, and BB; and q is a weighting factor given as $q=1/\sum_{j=1}^{n}2p_{j}\left(1-p_{j}\right)$, where $p_{j}$ is the second allele frequency of the *j-th* SNP marker.

### 2.4. Genetic Parameters

Genetic parameters were obtained with the average information restricted maximum likelihood procedure using a multi-trait animal model and the AIREMLF90 program of the BLUPF90 family [17].

Heritability estimates ($h_{a}^{2}$) for the CN fractions (α_S1_-, α_S2_-, β-, and κ-CN) and WP fractions (β-LG and α-LA), and for the total protein, casein (CN), and whey protein (WP) contents, proportions, and daily yields were determined as: $h_{a}^{2}=\frac{\sigma_{a}^{2}}{\sigma_{a}^{2}+\sigma_{e}^{2}}$, where $\sigma_{a}^{2}$ and $\sigma_{e}^{2}$ are the genetic and residual variances, respectively. The correlations among the milk protein fractions were calculated as: $r_{a}=\frac{{\hat{\sigma }}_{a_{m,n}}}{\sqrt{{\hat{\sigma }}_{m}^{2}\times {\hat{\sigma }}_{n}^{2}}}$. To assess the impact on animal rankings of expressing milk proteins qualitatively (%N) or quantitatively (g/100g M and g/d), we calculated the Spearman’s correlations among the genomic breeding values (GEBV) for each trait expressed in the three different ways (g/L, %N and g/d), and assessed the overlap among the top 5% of animals with the highest GEBV for each trait. The GEBV was calculated as the sum of the predicted SNP effects, as $GEBV_{i}=\;\sum_{j=1}^{k}M_{ik}{\hat{w}}_{k}$, where ${\hat{w}}_{k}$ is the effect of the *k*-*th* SNP marker, using the predictf90 program of the BLUPF90 family [17].

## 3. Results

### 3.1. Descriptive Statistics

The descriptive statistics for the contents, proportions and daily yields of the milk protein fractions (caseins and whey proteins), milk crude protein content, and milk production are shown in Table 1. The cows produced an average of 24.70 ± 7.68 kg milk. The average contents of the caseins were 9.41 ± 1.20 (α_S1_-CN), 11.79 ± 1.39 (β-CN), 3.47 ± 0.68 (κ-CN), and 3.37 ± 0.57 (α_S2_–CN) g/L. The average contents of the whey proteins were 3.19 ± 0.70 (β-LG), and 0.87 ± 0.18 (*α*–LA) g/L. The milk protein fraction proportion was 87.67 %N, with caseins accounting for 76.62 %N and whey protein for 11.05%N. As expected, the milk caseins with the highest proportions were β-CN and α_S1_-CN. The average proportions of the casein fractions were 32.22 ± 2.38 (α_S1_-CN), 25.71 ± 1.71 (β-CN), 9.52 ± 1.39 (κ-CN), and 9.17 ± 1.05 (α_S2_–CN) %N. The average proportions of the whey proteins β-LG and α-LA were 8.66 ± 1.47 and 2.39 ± 0.48 %N, respectively. Regarding daily yields, casein fractions secreted into the milk were 289.48 ± 81.02 (α_S1_-CN), 230.58 ± 72.18 (β-CN), 84.63 ± 27.76 (κ-CN), and 83.07 ± 26.95 (α_S2_–CN) g/d; whey fractions secreted into the milk were 78.04 ± 27.02 (β-LG), and 21.64 ± 8.71 (α-LA) g/d. With respect to the phenotypic values, the highest variability was observed for daily yields compared with proportions and contents.

### 3.2. Genetic Parameters

The heritability estimate for milk yield was low (0.12 ± 0.027; Table 2). The heritability estimate for total whey proteins was slightly higher than for total caseins (Table 2). The genetic variance component estimates for caseins (α_S1_-, α_S2_-, β- and к-CN) and whey proteins (α-LA and β-LG) expressed as contents, proportions and daily yields differed substantially, providing strong evidence for different genetic outcomes (Table 2). The most important differences were between casein and whey protein daily yields and their contents and proportions (Table 2). The heritability estimates for α-LA, whether for content, proportion or daily yield, were similar, with values of 0.21 ± 0.0074 (g/L), 0.23 ± 0.0081 (%N) and 0.23 ± 0.0078 (g/d), respectively. The heritability estimates for β-CN exhibited the largest differences, with values of 0.59 ± 0.0042 for content, 0.78 ± 0.0037 for proportion, and 0.22 ± 0.0025 for daily yield (Table 2).

Average heritability estimates for the milk protein fractions varied depending on whether they were expressed as contents, proportions or daily yields (Table 2). The highest heritability estimates were for β–CN content and proportion (h^2^ = 0.59 and h^2^ = 0.78, respectively), while the highest heritability for daily yield was for β-LG (h^2^ = 0.38). However, β-LG content and proportion had the same heritability estimate (h^2^ = 0.53), which was higher than that for daily yield. The heritability estimates for milk protein daily yield were around 46.13% lower than for milk protein content, and around 48.76% lower than for the proportion (Table 2). The heritability estimates for κ-CN, α_S1_-CN, and α_S2_-CN proportions were slightly higher than those for casein content.

### 3.3. Genetic Correlations

The genetic correlations for milk protein fraction contents (g/L) ranged from −0.48 (β-CN and κ-CN) to 0.74 (α_S2_-CN and α_S1_–CN; Table 3). Milk protein content exhibited weak to moderate negative correlations with milk yield, except for α_S1_–CN which was positively correlated (0.22). Low to moderate genetic correlations were found between the milk protein fraction proportions (%N) and crude protein (CP), which were positive for κ–CN (0.17) and β-LG (0.18), and negative for α_S1_–CN (−0.41), α_S2_–CN (−0.15), β–CN (−0.25) and α–LA (−0.50) (Table 3). Genetic correlations for milk protein fraction proportions (%N) ranged from −0.78 (β-CN and κ-CN) to 0.67 (α_S2_-CN and α–LA), and for daily yields they ranged from −0.30 (β-CN and κ-CN) to 0.85 (α_S2_-CN and α_S1_-CN; Table 3). High, positive genetic associations were found between α_S2_-CN and α_S1_-CN in terms of contents and daily yields, while moderate positive values were found for their proportions.

The estimated additive genetic correlations between β-CN and κ-CN were moderate and negative for contents and daily yields (−0.48 and −0.30, respectively), high and negative for proportions (−0.78; Table 3). The genetic correlations between the β-CN proportion and the α_S1_-CN, α_S2_-CN, and β-LG proportions were negative, but positive for daily yields. The β-CN content exhibited negative genetic correlations with the α_S1_-CN and α_S2_–CN contents, but a positive genetic correlation with β–LG content. The β-LG proportion exhibited low, negative genetic correlations with the β-CN and κ-CN proportions (−0.23 and −0.35, respectively), and low, positive genetic correlations with the α_S1_-CN and α_S2_-CN proportions (0.25 and 0.23, respectively). On the other hand, β-LG content and daily yield were positively associated with β-CN and κ–CN contents and daily yields, ranging from 0.17 to 0.37. However, the genetic correlations between β-LG daily yield and α_S1_-CN and α_S2_-CN daily yields were moderate (0.61 and 0.47, respectively), and were also moderate for contents (0.56 and 0.34, respectively).

The α-LA daily yield showed a null genetic correlation with *κ*-CN daily yield, and low genetic correlations with the κ-CN proportion (0.14) and content (0.21; Table 3). The genetic correlations between the whey protein α-LA and the caseins β-CN, α_S1_-CN, α_S2_-CN, and β-LG were weakly to moderately positive for proportions (%N), weakly negative to highly positive for contents (g/L), and moderately to highly positive for daily yields (g/d; Table 3).

### 3.4. Phenotypic Correlations

Phenotypic correlations among the milk protein fractions expressed in different ways tended to be in the same direction as the genetic correlations (Table 3). Overall, the phenotypic correlation estimates for milk protein fraction proportions were weaker than the genetic correlations but were higher for daily yields. All protein fraction contents had weak negative phenotypic correlations with milk yield, except for β-CN, which had the strongest correlation (−0.63). The estimated phenotypic correlations between milk yield and milk protein content tended to be in the same direction as the estimated genetic correlations, but lower, except for β–CN and β-LG (Table 3). Crude protein correlated negatively with the *β*-CN and *α*–LA proportions, but positively with α_S1_-CN and α_S2_–CN. Null phenotypic associations were observed between the β-CN and α-LA proportions, the κ-CN and α_S1_–CN proportions, the α_S1_-CN and α_S2_–CN proportions, and the β-CN and κ-CN contents (Table 3). We found the environmental correlations to be high among milk protein fraction daily yields, moderate among milk protein contents, and weak-moderate among milk protein proportions (Table S1).

### 3.5. Spearman’s Correlations

**Figure 1:**
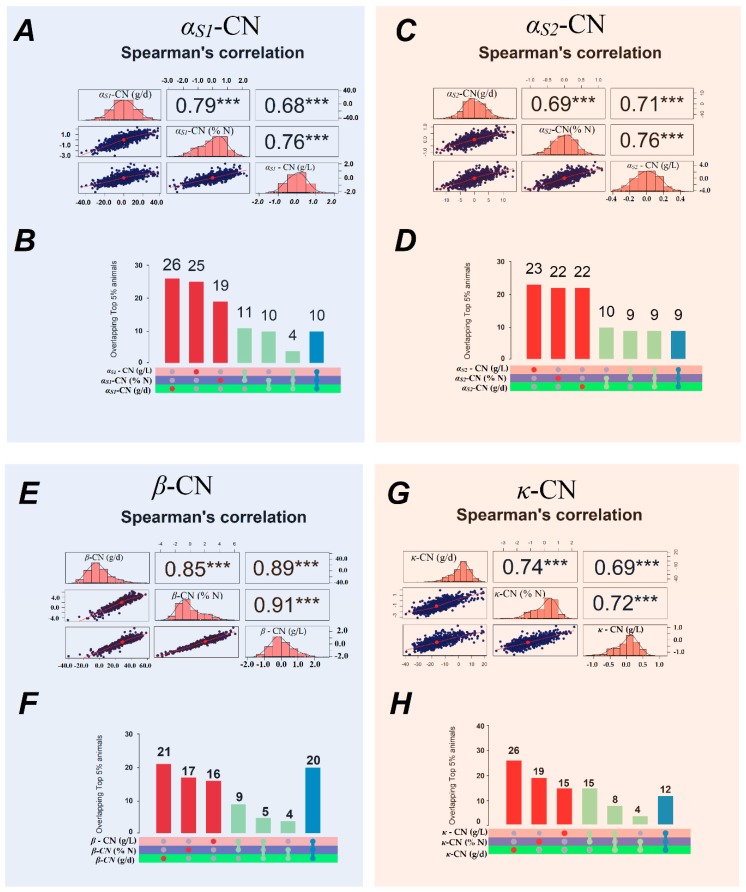
Genomic breeding value (GEBV) distributions (diagonal), Spearman’s correlations of GEBV (upper triangle), and scatter plots (lower triangle) for α_S1_-CN (**A**), α_S2_-CN (**C**), β-CN (**E**) and k-CN (**G**), specific to and shared across the 5% of animals (n = 50) with the highest GEBV for α_S1_-CN (**B**), α_S2_-CN (**D**), β-CN (**F**), and k-CN (**H**) contents (g/L), proportions (%N), and daily yields (g/d).

**Figure 2:**
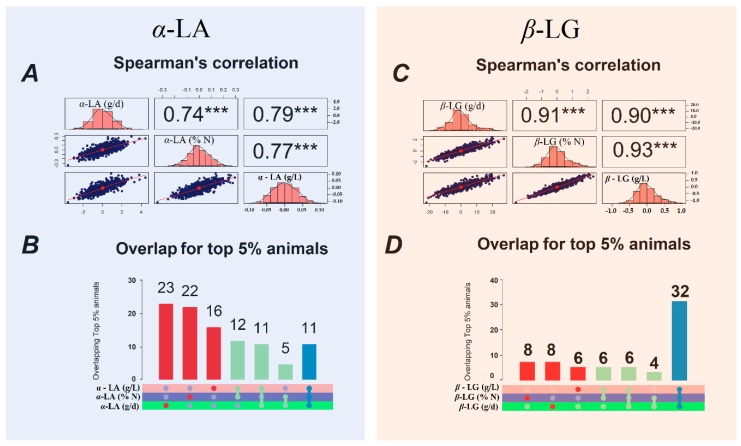
Genomic breeding value (GEBV) distributions (diagonal), Spearman’s correlations of GEBV (top triangle), and scatter plots (lower triangles) for α-LA (**A**) and β-LG (**C**), and the overlap between the 5% of animals (n = 50) with the highest GEBV for α-LA (**B**), and β-LG (**D**) contents (g/L), proportions (%N), and daily yields (g/d).

The rank correlations between the predicted genomic breeding values (GEBV) for the milk protein fractions expressed in different ways ranged from 0.68 (between κ-CN daily yield and content) to 0.93 (between β-LG proportion and content; Figure 1 and Figure 2). The Spearman’s correlation estimates show that alterations to the ranking of animals based on their GEBV for milk protein fractions are to be expected when these traits are differently expressed.

The Spearman’s correlations among the GEBV for the caseins expressed in different ways varied from medium to high (Figure 1A,C,E,G). These results show that animals are often reclassified according to how the caseins are expressed, which results in different coincidence rates between the top 5% of animals by GEBV (Figure 1B,D,F,H). Among the milk proteins, κ-CN was most frequently reclassified, and exhibited a lower number of overlaps among the top 5% of animals by GEBV (Figure 1D). In general, the top animals were less frequently re-ranked when milk proteins were expressed as contents and proportions than when they were expressed as daily yields (Figure 1).

The whey protein β-LG exhibited the highest Spearman’s correlation coefficient (r ≥ 0.90) among the GEBV (Figure 2). These results show there is a high overlap among the top 5% animals for β-LG and a lower rate of re-ranking (Figure 2). On the other hand, the Spearman’s correlations for α-LA show that the animals are often re-ranked according to whether it is expressed as content, proportion or daily yield (Figure 2).

## 4. Discussion

We carried out this study in order to investigate potential differences in the genetic parameters of milk protein fractions (casein and whey proteins) according to whether they are measured as contents, proportions or daily yields. Milk protein composition is affected mainly by genetic factors, as well as by non-genetic factors. In this study, the caseins α_S1_-CN and β-CN were found in higher proportions than *α_S2_*-CN and *k*-CN, representing approximately 75.61% of the total casein content, in agreement with various studies on different dairy breeds [5,10,18,19]. Among the whey protein fractions, we found β-LG in amounts approximately 3.5 times that of α-LA. This result is in agreement with data from previous studies [9,19,20].

### 4.1. Genetic Parameters

The heritability estimates for casein and whey protein proportions indicate that a considerable part of the variation for these traits is influenced by the additive genetic effect (Table 2). Milk protein fraction daily yields exhibited lower heritability estimates than the proportions, due to the low heritability of single-test-day milk yields, and genetic progress would therefore be particularly slow for these traits. The higher heritabilities observed for k-CN and β-CN proportions might be due to the fact that single protein fractions are highly regulated by the *CSN2* - *CSN3* haplotype [21]. Our findings are in agreement with those of Pegolo et al. [9] and Bonfatti et al. [3], who reported that the qualitative data obtained from milk protein fraction proportions resulted in higher estimates.

The lowest heritability estimates for milk protein proportions were for α-LA (0.23), while the highest were for β-CN (0.78), in agreement with previous data [9]. Schopen et al. [5], however, obtained a higher heritability estimate for β-LG (0.80), and a lower one for β-CN (0.25). These differences might be due to different breeds and allele frequencies affecting the milk protein content [22]. Some differences were found among the heritability estimates for the major caseins (from 0.78 for β-CN to 0.29 for α_S2_-CN). These heritability estimates are directly affected by the genetic link between the CN haplotype and the proportions of these fractions [23]. Bonfatti et al. [3] reported a similar heritability estimate to ours of 0.28 for α_S2_-CN, and Pegolo et al. [9] obtained a slightly one (0.36). In contrast, Schopen et al. [5] reported a much higher heritability for α_S2_-CN (0.73) in Dutch Holstein-Friesians.

The moderate heritability estimates for α-LA content, proportion and daily yield (Table 2) were similar to the values reported by Gebreyesus et al. [8], confirming a combination of substantial genetic and environmental effects on the expression of this trait. Higher heritability estimates for α–LA as a proportion have been reported in Dutch Holstein-Friesians (0.55) [5], in French Holstein (0.44), Montbéliarde (0.72) and Normande (0.53) [10], and in Danish Holsteins (0.40) and Danish Jerseys (0.44) [24] breeds, although with a standard error higher than ours. The heritability obtained in our study for β-LG as a proportion was moderate and consistent with previous results reported by Buitenhuis et al. [24] for Danish Holstein (0.58), Gebreyesus et al. [8] for Danish Holstein (0.56), Pegolo et al. [9] for Italian Brown Swiss (0.558) and Sanchez et al. [10] for French Holstein (0.71), Montbéliarde (0.79) and Normande (0.72) breeds. On the other hand, Schopen et al. [5] obtained a higher mean heritability estimate (0.80), and Bonfatti et al. [3] a lower mean heritability estimate (0.34) than we did. These variations in heritability estimates across different studies may be partly due to different breeds, sample sizes, and statistical models used.

Differences in the genetic components were observed according to whether the milk proteins were measured as contents, proportions or daily yields (Table 2), which suggests that from a genetic perspective they could be considered as different traits. Measuring milk protein fractions in terms of daily yields seems to imply that their phenotypic expression is highly influenced by environmental factors. The lower heritability estimates for α-LA could be partly due to greater environmental effects on its relative concentrations compared with other fractions. Our heritability results for casein and whey protein contents were higher than those reported by Bonfatti et al. [3], which ranged from 0.11 (α-LA) to 0.53 (k-CN). Sanchez et al. [10] found lower heritability estimates for α_S1_-CN, β-CN, and k-CN in the Normande (NO) and Holstein (HO) breeds, although they obtained higher heritabilities for the Montbéliarde breed.

Overall, the heritability estimates for milk protein fraction proportions were higher than those for contents and daily yields, which suggests that qualitative measures of these traits capture the influence of milk protein genotypes more effectively [18,23]. This seems to indicate that predictions of expected responses to selection for major casein and whey proteins might differ according to whether the milk proteins are expressed qualitatively or quantitatively. Therefore, a greater potential for genetic gain is expected when milk proteins are measured as proportions, which is supported by higher heritability estimates and higher predictive ability measured by the Pearson’s correlation among the GEBV and corrected phenotype (Supplementary information Figure S3). The observed differences in the predictive ability for milk protein fractions expressed in different ways (g/L, %N and g/d) may be explained by different biological perspectives related to their genetic variation. Thus, considering milk proteins expressed as daily yields, which accounts for the cows‘ milk production levels, might reflect the combination of genetic and environmental factors.

### 4.2. Genetic and Phenotypic Correlations

The importance of estimating genetic and phenotypic correlations between milk protein fraction contents, proportions and daily yields is to gain a better understanding of how caseins and whey proteins might respond to selection in dairy cattle. The phenotypic correlations among milk protein fraction daily yields were higher than those among proportions and contents, providing further evidence of the greater effect of environmental factors. The differences in the phenotypic correlations according to whether the milk proteins were expressed as contents, proportions or daily yields may be explained by differences in the contributions of genetic and environmental effects. However, the same trend was observed in the genetic and phenotypic correlations for milk protein fractions. According to Cheverud [25] and Sodini et al. [26], similar directions for phenotypic and genetic correlations could be due to environmental effects acting in the same direction as the genetic effects.

The genetic relationships among the milk protein fraction contents, proportions and daily yields were expected, since caseins are clustered on BTA6 within a 250 kb region [27], and caseins and whey proteins have a common biological regulatory mechanism controlling their synthesis [9,28]. Indeed, the genomic region controlling casein and whey protein expression exhibits a considerable linkage disequilibrium [29]. There is a high positive genetic correlation between milk yield and milk protein content [30]. We found a lower positive genetic correlation between milk yield and the content of α_S1_–CN, one of the major milk protein fractions together with β–CN. On the other hand, we found a moderate negative correlation between milk yield and β–CN, and weak negative correlations between milk yield and the other milk protein fraction contents. These results indicate that selection for milk yield has little effect on the contents of individual milk proteins. A similar pattern was observed regarding the genetic correlations between crude protein and milk protein proportions, except for the correlation between crude protein and α-LA and α_S1_-CN, which was moderate and negative in both cases. Selection for milk protein content is therefore expected to have a small to moderate effect on milk protein composition.

β-CN correlated negatively with κ-CN, as proportions (r = −0.78) and as daily yields and contents (r = −0.30), in agreement with Bonfatti et al. [3] and Sanchez et al. [10], who determined milk proteins as qualitative traits. On the other hand, Bonfatti et al. [3] and Schopen et al. [5] found null genetic correlations between β-CN and κ-CN contents in Dutch Holstein-Friesians (r = −0.04) and Simmentals (r = 0.08). Furthermore, Bonfatti et al. [3] looked at *CSN2* - *CSN3* haplotypes and β-LG genotype effects and found positive genetic correlations between β-CN and κ-CN (r = 0.66). This further confirms that milk protein fractions have common regulatory mechanism controlling their synthesis. The negative correlation observed between β-CN and κ-CN could be ascribed to the existence of some compensatory regulatory mechanisms, due to the remarkably strong linkage among genes coding for these milk protein variants.

We found negative genetic associations between β-CN and α_S1_-CN, and between β-CN and α_S2_-CN when expressed as proportions (r = −0.70 and −0.43, respectively) and as contents (r = −0.30 and −0.23, respectively), but positive correlations when expressed as daily yields (r = 0.42 and 0.38, respectively). These results are in agreement with the literature [3,8]. However, other authors obtained null estimates between β-CN and α_S1_-CN [5]. It has been suggested that increasing the β-CN concentration in milk is associated with a decrease in the α_s1_-CN content, which seems to be caused by a competitive synthesis effect [18,21,22,23,31]. However, expressing milk proteins as proportions tend to generate negative covariances among them, especially in the case of β-CN and α_S1_-CN which are the major milk proteins in bovine milk.

The moderate and high genetic correlations among α_S1_-CN, α_S2_-CN, and κ–CN proportions, contents and daily yields indicate that using sires selected for these traits should result in progenies able to produce milk enriched in these casein fractions. The κ-CN fraction has a strong positive effect on milk coagulation, curd firming, and the syneresis process; similarly, the α_S1_-CN fraction in milk has a favorable effect on curd firmness. On the other hand, the α_S2_-CN fraction seems to have the opposite effect to α_S1_-CN, since it delays milk gelation, and reduces the maximum curd firmness reached within 90 min as well as syneresis [20]. Sanchez et al. [10] also reported positive genetic associations among α_S1_-CN, α_S2_-CN, and κ–CN contents and proportions, with values ranging from 0.84 to 0.98. Several studies have shown that differences in the promoter region of *CSN3* may give rise to different effects on the content of the α_S1_-CN and α_S2_-CN fractions [22,23,31].

The genetic correlations between the proportion of the major whey protein β-LG and the casein fractions β-CN and к-CN were negative (−0.23 and −0.35, respectively). Regarding daily yields and contents, the genetic associations between β-LG and the casein fractions β-CN and к-CN were positive, with values ranging from 0.17 to 0.37. Sanchez et al. [10] reported negative correlations between the proportion of β-LG and the proportions of β-CN and к-CN ranging from −0.20 to −0.75 in three breeds; with regard to contents, however, the same authors obtained positive associations, with values ranging from 0.07 to 0.58. Heck et al. [21] and Bobe et al. [23] observed that β-LG variants affected determination of its proportion in milk, and also affected β-CN and k-CN proportions of the total milk protein. These correlations indicate that selection for lower proportions of β-LG should increase the proportion of k-CN in milk, with positive effects on milk coagulation [18,20,32]. Highly significant interactions between β-CN, κ-CN and β-LG have been reported in literature which could be explained by the existence of epistatic effects between the genes coding for these proteins [33].

We obtained positive genetic associations between β-LG and α_S1_-CN, and between β-LG and α_S2_-CN. Bonfatti et al. [3] found a null genetic correlation between the proportion of β-LG and the proportions of α_S1_-CN and α_S2_–CN; however, these authors found low to moderate correlations between them in terms of contents (r = 0.26 and r = 0.35, respectively). Regarding the *CSN2* - *CSN3* haplotypes and β-LG genotype effects, we obtained higher estimates, which provides further evidence that β-LG controls the proportion of α_S1_-CN in milk [3]. In agreement with this, several studies have shown that the haplotypes with positive effects on the β-LG percentage reduce the α_S1_-CN percentage in milk [18,21,22,23].

We found a null association between α-LA and k-CN daily yields (r = 0.07), and a weak negative association between α-LA and β-CN contents (r = −0.16). In our study, population, we observed positive genetic associations ranging from low-moderate to high between the α - LA content and the k - CN, α_S1_ - CN, α_S2_ - CN, and β – LG contents, although Bonfatti et al. [3] reported lower estimates. On the other hand, Gebreyesus et al. [8] found a negative association between α-LA and α*_S_*_1_-CN (*r* = −0.32). Nevertheless, Sanchez et al. [10] reported a low association between *α*-LA and β–CN (0.18), and a high association between α-LA and k–CN (0.80). Moreover, we found moderate associations between α-LA and β–*LG* expressed as contents, proportions and daily yields, while several authors found genetic correlations ranging from negative (r = −0.57) to low positive (r = 0.27) [3,5,8,10].

### 4.3. Spearman’s Correlations

The Spearman’s rank correlations between breeding values calculated with multi-trait models for casein and whey protein contents, proportions and daily yields were significant for α_S1_-CN, α_S2_-CN, к-CN, and α-LA. In general, the correlations among milk protein fraction proportions and contents were higher than among daily yields (Figure 1 and Figure 2). The rank correlations for casein fractions ranged from moderate (r = 0.68) to high (r = 0.91), indicating some differences in the animals selected based on GEBVs. The highest rank correlation was between the β-*C*N proportion and content (r = 0.91), followed by that between daily yield and content (r = 0.89). On the other hand, the greatest frequency of re-ranking of animals was observed for the α_S2_-CN and α - LA fractions, based on the Spearman’s correlations and the overlap between the top 5% of animals.

The results of the rank correlation lead us to speculate that expressing milk proteins in different ways has an impact on the outcome of selection strategies in dairy cattle. It is of note that the lower Spearman’s correlations for α_S1_-CN, α_S2_-CN and α-LA might be due to an effect of unknown environmental and non-additive factors leading to the differences in the ranking of animals for these traits according to whether they are expressed as proportions, contents or daily yields.

## 5. Conclusions

Our study shows that expressing milk protein fractions variously as proportions, contents or daily yields can lead to different genetic outcomes. In particular, protein fraction proportions may be associated with a more effective response to selection than contents or daily yields. The differences in the patterns of genetic and phenotypic correlations for caseins and whey proteins suggest that different ways of expressing them capture different biological processes. The Spearman’s correlations and coincidence rates indicate substantial differences in genetic variation, and major effects as a result of re-ranking the animals. This information will be useful in developing breeding programs aimed at improving milk quality and cheese-making ability. Before that; however, the occurrence of possible adverse effects of selection based on milk protein composition on cow’s fitness, health, and welfare should be further investigated. Moreover, for large-scale phenotyping of milk protein fractions, fast and cost-effective tools such as Fourier transform infrared (FT-IR) spectroscopy might represent an effective alternative to the gold standard analytical methodology for routinely measuring these traits at a population level for breeding purposes.

## Figures and Tables

**Table 1:** Descriptive statistics for milk production, somatic cell scores, and milk proteins in Brown Swiss cattle.

Trait^1^	N	Mean	Min	Max	SD	V.C.
*Milk yield (kg/d)*	981	24.87	5.91	45.33	7.28	29.27
*Protein fraction contents (g/L)*						
Major protein fractions	983	32.07	22.90	42.21	3.49	10.88
Major caseins	983	28.03	19.94	36.90	2.97	10.59
α_S1_-CN	987	9.41	5.80	12.99	1.20	12.75
α_S2_-CN	986	3.37	1.98	5.11	0.57	16.91
β-CN	985	11.79	8.11	16.02	1.39	11.79
κ-CN	984	3.47	1.36	5.53	0.68	19.59
Major whey proteins	981	4.05	1.76	6.26	0.77	19.01
α-LA	982	0.87	0.38	1.42	0.18	20.69
β-LG	988	3.19	1.15	5.27	0.70	21.94
*Crude protein (g/L)*	985	36.60	25.80	48.40	4.10	10.79
*Protein fraction proportions (%N)*						
Major protein fractions	980	87.67	81.77	93.55	2.25	2.57
Major caseins	981	76.62	73.27	79.83	1.24	1.62
α_S1_-CN	985	25.71	20.55	30.91	1.71	6.65
α_S2_-CN	979	9.17	6.26	12.58	1.05	11.45
β-CN	976	32.22	25.97	39.41	2.38	7.39
κ-CN	985	9.52	5.02	13.52	1.39	14.60
Major whey proteins	979	11.05	6.69	15.46	1.52	13.76
α-LA	983	2.39	1.03	3.81	0.48	20.08
β-LG	978	8.66	4.08	13.37	1.47	16.97
*Protein fraction daily yields (g/d)*						
Major protein fractions	986	787.44	218.68	1370.19	225.67	28.66
Major caseins	984	687.76	191.33	847.99	95.75	13.92
α_S1_-CN	983	230.58	46.07	431.04	72.18	31.30
α_S2_-CN	987	83.07	15.73	163.49	26.95	32.44
β-CN	984	289.48	75.78	543.91	81.02	27.99
κ-CN	984	84.63	11.42	169.78	27.76	32.80
Major whey proteins	983	99.68	21.36	190.73	33.11	33.21
α-LA	983	21.64	4.01	47.97	8.71	40.25
β-LG	985	78.04	15.11	159.48	27.02	34.62

CN: casein; β-LG: β-lactoglobulin; α-LA: α-lactalbumin; g/d: grams of protein secreted per day; %N: percentage of nitrogen; g/L: grams per L of milk; Major protein fractions: sum of the whey proteins and caseins; Major caseins: sum of the casein fractions (β-CN + κ-CN + α_S1_-CN + α_S2_-CN); Major whey proteins: sum of the whey protein fractions (β-LG + α-LA); V.C.: variance coefficient.

**Table 2:** Estimates (and standard errors) of genetic ($\sigma_{g}^{2}$), and phenotypic ($\sigma_{p}^{2}$) variances, and heritabilities ($h^{2}$) for milk production and milk protein fractions.

Trait	$\sigma_{g}^{2}$	$\sigma_{p}^{2}$	$h^{2}$
*Milk yield (kg/d)*	2.79 ^(0.892)^	22.09 ^(1.256)^	0.12 ^(0.033)^
*Protein fraction contents (g/L)*			
Major protein fractions	2.41 ^(0.020)^	6.07 ^(0.094)^	0.39 ^(0.007)^
Major caseins	1.68 ^(0.045)^	4.41 ^(0.088)^	0.38 ^(0.009)^
α_S1_-CN	0.51 ^(0.002)^	0.92 ^(0.007)^	0.55 ^(0.001)^
α_S2_-CN	0.05 ^(0.001)^	0.18 ^(0.009)^	0.27 ^(0.002)^
β-CN	0.64 ^(0.002)^	1.08 ^(0.005)^	0.59 ^(0.004)^
κ-CN	0.18 ^(0.007)^	0.33 ^(0.015)^	0.55 ^(0.005)^
Major whey proteins	0.12 ^(0.003)^	0.29 ^(0.012)^	0.43 ^(0.005)^
α-LA	0.003 ^(0.000)*^	0.014 ^(0.000)*^	0.21 ^(0.007)^
β-LG	0.12 ^(0.001)^	0.24 ^(0.008)^	0.53 ^(0.006)^
*Crude protein (g/L)*	0.36 ^(0.007)^	0.82 ^(0.06)^	0.44 ^(0.06)^
*Protein fraction proportions (%N)*			
Major protein fractions	0.84 ^(0.013)^	2.07 ^(0.023)^	0.40 ^(0.009)^
Major caseins	0.37 ^(0.054)^	1.01 ^(0.068)^	0.37 ^(0.009)^
α_S1_-CN	1.20 ^(0.003)^	2.06 ^(0.066)^	0.58 ^(0.009)^
α_S2_-CN	0.23 ^(0.002)^	0.80 ^(0.018)^	0.29 ^(0.008)^
β-CN	3.67 ^(0.004)^	4.69 ^(0.092)^	0.78 ^(0.003)^
κ-CN	1.10 ^(0.009)^	1.83 ^(0.081)^	0.60 ^(0.006)^
Major whey proteins	0.56 ^(0.010)^	1.29 ^(0.082)^	0.43 ^(0.011)^
α-LA	0.02 ^(0.003)^	0.11 ^(0.022)^	0.23 ^(0.008)^
β-LG	0.63 ^(0.003)^	1.20 ^(0.045)^	0.53 ^(0.005)^
*Protein fraction daily yields (g/d)*			
Major protein fractions	283.60 ^(1.206)^	2015.45 ^(1.348)^	0.15 ^(0.004)^
Major caseins	207.47 ^(0.985)^	1320.91 ^(1.055)^	0.14 ^(0.007)^
α_S1_-CN	285.35 ^(2.578)^	1284.45 ^(17.086)^	0.25 ^(0.002)^
α_S2_-CN	52.60 ^(0.231)^	258.12 ^(6.345)^	0.20 ^(0.009)^
β-CN	521.10 ^(2.739)^	2334.14 ^(16.226)^	0.22 ^(0.002)^
κ-CN	105.65 ^(0.380)^	357.63 ^(3.469)^	0.29 ^(0.001)^
Major whey proteins	90.81 ^(0.029)^	340.66 ^(0.832)^	0.21 ^(0.007)^
α-LA	5.45 ^(0.020)^	23.47 ^(0.679)^	0.23 ^(0.007)^
β-LG	97.09 ^(0.353)^	256.29 ^(4.904)^	0.38 ^(0.003)^

CN: casein; β-LG: β-lactoglobulin; *α*-LA: α-lactalbumin; g/d: grams of protein secreted per day; %N: percentage of nitrogen; g/L: grams per L of milk; Major protein fractions: sum of the whey proteins and caseins; Major whey proteins: sum of the whey protein fractions (β-LG + α-LA); Major caseins: sum of the casein fractions (β-CN + κ-CN + α_S1_-CN + α_S2_-CN); ^(0.000)*^: a standard error below 0.0001.

**Table 3:** Estimates of genetic correlations (above diagonal) and phenotypic correlations (below diagonal; standard error) among milk protein fractions.

	*Protein Fraction Contents (g/L)*						
***Title***	**MY**	**α_S1_-CN**	**α_S2_-CN**	**β-CN**	**κ-CN**	α-LA	β-LG
MY	-	0.27 ^(0.007)^	−0.31 ^(0.004)^	−0.42 ^(0.006)^	−0.27 ^(0.008)^	−0.20 ^(0.005)^	−0.15 ^(0.005)^
α_S1_-CN	0.02 ^(0.004)^	-	0.74 ^(0.002)^	−0.30 ^(0.002)^	0.55 ^(0.001)^	0.44 ^(0.001)^	0.56 ^(0.001)^
α_S2_-CN	−0.29 ^(0.001)^	0.55 ^(0.006)^	-	−0.23 ^(0.001)^	0.34 ^(0.002)^	0.71 ^(0.001)^	0.34 ^(0.004)^
β-CN	−0.63 ^(0.004)^	−0.36 ^(0.002)^	−0.25 ^(0.002)^	-	−0.48 ^(0.004)^	−0.12 ^(0.002)^	0.17 ^(0.003)^
κ-CN	−0.14 ^(0.002)^	0.44 ^(0.004)^	0.26 ^(0.005)^	−0.03 ^(0.002)^	-	0.21 ^(0.003)^	0.33 ^(0.006)^
α-LA	−0.04 ^(0.001)^	0.33 ^(0.002)^	0.42 ^(0.003)^	0.19 ^(0.002)^	−0.11 ^(0.002)^	-	0.42 ^(0.001)^
β-LG	−0.26 ^(0.002)^	0.55 ^(0.002)^	0.45 ^(0.004)^	0.30 ^(0.003)^	0.32 ^(0.004)^	0.38 ^(0.002)^	-
*Title*	*Protein Fraction Proportions (%N)*						
	CP	α_S1_-CN	α_S2_-CN	β-CN	κ-CN	α-LA	β-LG
CP	-	−0.41 ^(0.002)^	−0.15 ^(0.003)^	−0.25 ^(0.002)^	0.17 ^(0.003)^	−0.50 ^(0.004)^	0.18 ^(0.002)^
α_S1_-CN	0.16 ^(0.021)^	-	0.59 ^(0.001)^	−0.70 ^(0.005)^	0.56 ^(0.001)^	0.38 ^(0.001)^	0.25 ^(0.003)^
α_S2_-CN	0.07 ^(0.005)^	0.09 ^(0.005)^	-	−0.43 ^(0.002)^	0.36 ^(0.002)^	0.67 ^(0.002)^	0.23 ^(0.001)^
β-CN	−0.20 ^(0.003)^	−0.58 ^(0.005)^	−0.35 ^(0.008)^	-	−0.78 ^(0.001)^	0.20 ^(0.002)^	−0.23 ^(0.002)^
κ-CN	0.09 ^(0.005)^	0.08 ^(0.003)^	0.11 ^(0.007)^	−0.57 ^(0.004)^	-	0.14 ^(0.001)^	−0.35 ^(0.002)^
α-LA	−0.25 ^(0.001)^	0.05 ^(0.002)^	0.25 ^(0.002)^	0.04 ^(0.009)^	−0.13 ^(0.005)^	-	0.40 ^(0.002)^
β-LG	0.09 ^(0.002)^	0.18 ^(0.002)^	0.16 ^(0.003)^	−0.18 ^(0.006)^	−0.02 ^(0.005)^	0.24 ^(0.003)^	-
*Protein Fraction Daily Yields (g/d)*							
		α_S1_-CN	α_S2_-CN	β-CN	κ-CN	α-LA	β-LG
α_S1_-CN	-	-	0.85 ^(0.006)^	0.42 ^(0.001)^	0.51 ^(0.004)^	0.63 ^(0.020)^	0.61 ^(0.008)^
α_S2_-CN	-	0.84 ^(0.008)^	-	0.38 ^(0.001)^	0.39 ^(0.003)^	0.70 ^(0.012)^	0.47 ^(0.005)^
β-CN	-	0.84 ^(0.002)^	0.77 ^(0.003)^	-	−0.30 ^(0.003)^	0.45 ^(0.018)^	0.30 ^(0.005)^
κ-CN	-	0.75 ^(0.004)^	0.63 ^(0.008)^	−0.61 ^(0.003)^	-	0.07 ^(0.009)^	0.37 ^(0.001)^
α-LA	-	0.77 ^(0.026)^	0.76 ^(0.009)^	0.74 ^(0.009)^	0.57 ^(0.010)^	-	0.58 ^(0.030)^
β-LG	-	0.79 ^(0.002)^	0.73 ^(0.007)^	0.72 ^(0.002)^	0.64 ^(0.005)^	0.71 ^(0.004)^	-

*CP*: Crude protein (g/L); *MY*: Milk yield (kg/d); CN: casein; β-LG: β-lactoglobulin; α-LA: α-lactalbumin; g/d: grams of protein secreted per day; %N: percentage of nitrogen; g/L: grams per L of milk.

## Supplementary Materials

The following are available online at https://www.mdpi.com/2076-2615/10/2/336/s1, Figure S1: Boxplot of the phenotypic values for milk protein fractions casein fractions (α_S1_, α_S2_, β, and κ - CN) and whey proteins (β - LG and α - LA) expressed as (i) gram secreted per day (g/d); (ii) percentage of total milk nitrogen content (%N) and (iii) gram/L of milk (g/L) after phenotypic control. Figure S2 – (A) Number of k clusters to identify the population structure and (B) A principal components analysis revealed two breeding subgroups of Brown Swiss cattle based on the genomic kinship coefficient. Table S1: Estimates of environmental correlation for milk protein fraction estimated as a percentage of total milk nitrogen (N) content using genomic information, Figure S3: Predictive ability assessed in the multi-trait model by Pearson’s correlations between genomic breeding values (GEBV) and adjusted phenotypes (y*) for milk protein fractions contents (g/L), proportions (%N) and daily yields (g/d).
