# Supplementary material for: Genomic Analysis of Milk Protein Fractions in Brown Swiss Cattle
Source: Animals (Basel). 2020 Feb 20;10(2):336. doi: 10.3390/ani10020336 (PMC7070934; doi:10.3390/ani10020336)
Supplement: Supplementary file 1 [file animals-10-00336-s001.pdf]

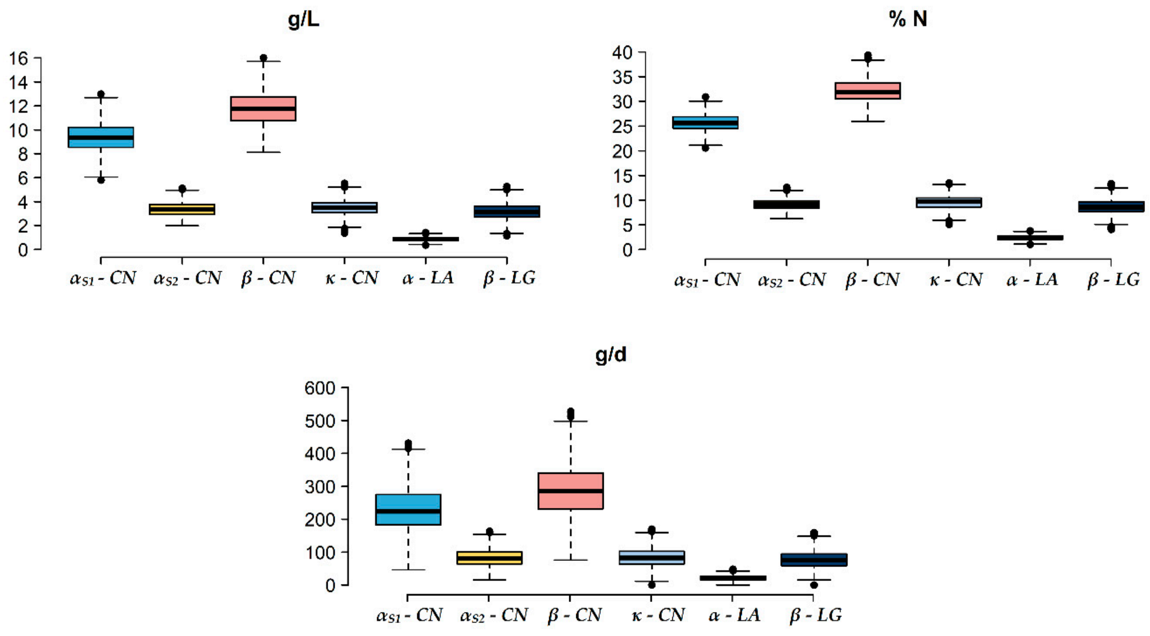

Figure S1. Boxplot of the phenotypic values for milk protein fractions casein fractions (α_{S1}, α_{S2}, β, and κ - CN) and whey proteins (α – LA and β - LG) expressed as i) gram per litter of milk (g/L); ii) percentage of total milk nitrogen content (%N) and iii) gram secreted per day (g/d) after phenotypic control.

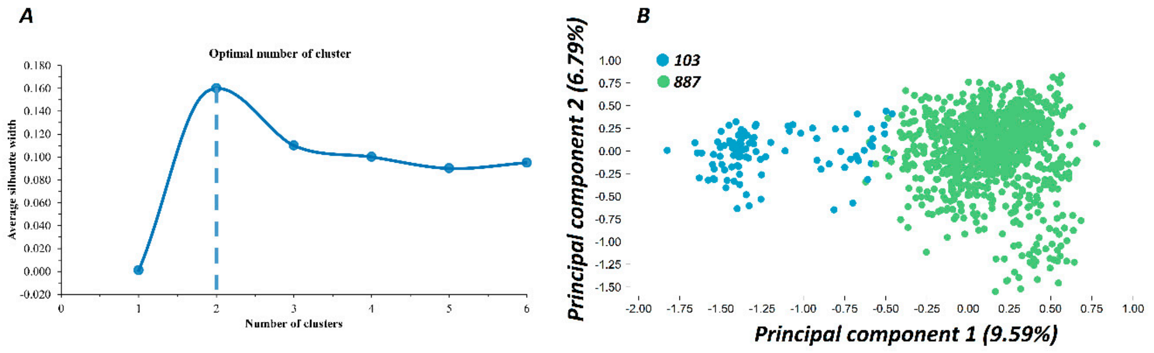

Figure S2. (**A**) Number of k clusters to identify the population structure and (**B**) A principal components analysis revealed two breeding subgroups of Brown Swiss cattle based on the genomic kinship coefficient.

Table S1. Estimates of environmental correlation for milk protein fractions using genomic information.

			Protein fractions contents (g/L)				
	MY	α_{S1}-CN	α_{S2}-CN	β-CN	κ-CN	α-LA	β-LG
MY	-	-0.36 (0.005)	-0.27 (0.003)	-0.25 (0.005)	-0.20 (0.001)	-0.03 (0.003)	-0.32 (0.005)
α_{S1}-CN		-	0.47 (0.050)	0.63 (0.045)	0.31 (0.055)	0.24 (0.066)	0.56 (0.033)
α_{S2}-CN			-	0.50 (0.045)	0.23 (0.071)	0.33 (0.071)	0.55 (0.043)
β-CN				-	-0.48 (0.031)	0.27 (0.046)	0.44 (0.045)
κ-CN					-	-0.24 (0.032)	0.31 (0.028)
α-LA						-	0.39 (0.037)
β-LG							-
			Protein fractions proportion (%N)				
	CP	α_{S1}-CN	α_{S2}-CN	β-CN	κ-CN	α-LA	β-LG
CP	-	0.04 (0.002)	0.15 (0.001)	-0.21 (0.006)	0.08 (0.009)	-0.10 (0.008)	0.05 (0.005)
α_{S1}-CN		-	0.23 (0.077)	-0.19 (0.012)	0.38 (0.063)	0.02 (0.036)	0.13 (0.060)
α_{S2}-CN			-	-0.29 (0.014)	0.27 (0.046)	0.15 (0.023)	0.29 (0.053)
β-CN				-	-0.15 (0.012)	0.04 (0.048)	-0.19 (0.020)
κ-CN					-	-0.01 (0.011)	-0.12 (0.026)
α-LA						-	0.28 (0.056)
β-LG							-
			Protein fractions daily yield (g/d)				
	-	α_{S1}-CN	α_{S2}-CN	β-CN	κ-CN	α-LA	β-LG
α_{S1}-CN	-	-	0.84 (0.004)	0.93 (0.015)	0.82 (0.003)	0.79 (0.012)	0.85 (0.003)
α_{S2}-CN			-	0.83 (0.019)	0.69 (0.005)	0.73 (0.009)	0.81 (0.010)
β-CN				-	-0.83 (0.029)	0.79 (0.009)	0.83 (0.076)
κ-CN					-	0.70 (0.004)	0.73 (0.003)
α-LA						-	0.76 (0.017)
β-LG							-

CP: Crude protein (g/L); *MY*: Milk yield (kg/d); CN: Casein; *β*-LG: β - lactoglobulin; *α*-LA: α - lactalbumin; g/d: Grams of protein secreted per day; %N: Percentage of nitrogen; g/L: Grams per L of milk.

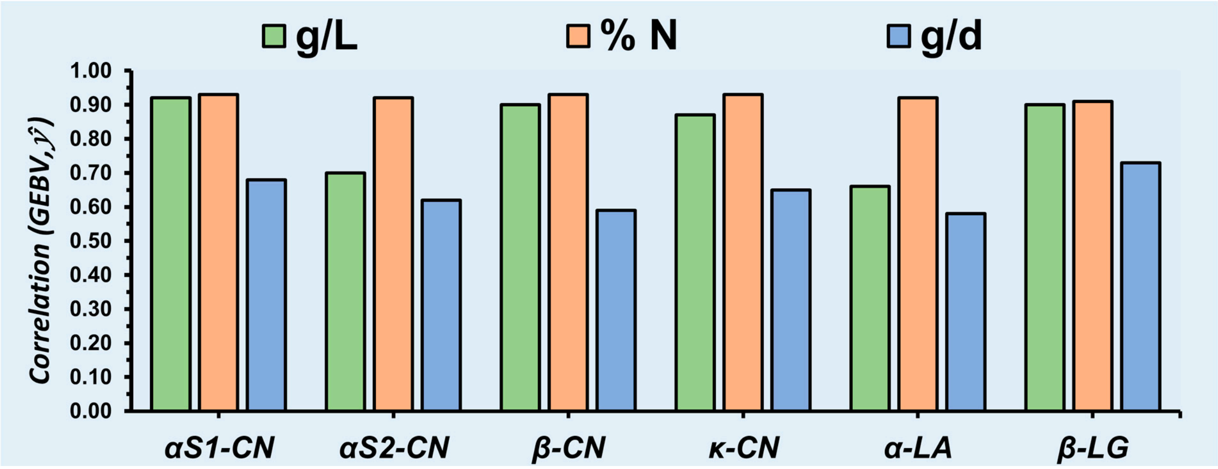

Figure S3. Predictive ability assessed in the multi-trait model by Pearson's correlations between genomic breeding values (GEBV) and adjusted phenotypes (y*) for milk protein fractions expressed as grams per liter of milk (g/L), percentage of nitrogen (%N) and grams per day (g/d).
